# Illegal Dumping Sites in Bloemfontein, South Africa: Respiratory Symptoms, Risk Factors, and Community Perspectives

**DOI:** 10.3390/ijerph22050685

**Published:** 2025-04-25

**Authors:** Botle Maluleka, Phoka C. Rathebe, Busisiwe Shezi

**Affiliations:** 1 Department of Environmental Health, Faculty of Health Sciences, University of Johannesburg, Johannesburg 2094, South Africa; buyangaphi88@gmail.com (B.M.); prathebe@uj.ac.za (P.C.R.); 2 Discipline of Occupational and Environmental Health, School of Nursing and Public Health, University of KwaZulu-Natal, Durban 4091, South Africa; 3 Environment and Health Research Unit, South African Medical Research Council, Durban 4091, South Africa

**Keywords:** illegal dumping, respiratory symptoms, proximity, health outcomes, perceptions, public awareness, South Africa

## Abstract

Illegal waste dumping is a significant global issue, particularly in low- and middle-income countries such as South Africa. This study aimed to investigate the risk factors for acute respiratory symptoms among residents living near illegal domestic waste dumping sites. The study also explored community perceptions regarding illegal dumping and its health effects. This cross-sectional study was conducted in Rocklands, Free State, South Africa, with 200 participants: 117 living within 0–5 km of a dumping site and 83 residing ≥5–10 km away. Data were collected using a structured questionnaire. Generalized linear models were employed to explore the relationship between proximity to illegal dumping sites and respiratory symptoms, adjusting progressively for confounders in successive models. Respiratory symptoms were more prevalent among those within 5 km of illegal dumping sites: cough (48.0% vs. 27.0%), shortness of breath (23.0% vs. 18.0%), wheezing (20.0% vs. 7.0%), and chest tightness (20.0% vs. 7.0%). Proximity was significantly associated with wheezing (PR: 2.77; 95% CI: 1.10–6.98) and chest tightness (PR: 2.86; 95% CI: 1.19–6.84). Community-driven initiatives, such as awareness campaigns and recycling, were strongly supported as solutions. These findings highlight the need for education on waste management. Collaborative efforts are essential to reduce illegal dumping and improve waste management.

## 1. Introduction

Illegal waste disposal is a global crisis affecting countries worldwide, particularly in low- and middle-income countries (LMICs). According to the World Health Organization (WHO), over 2 billion tons of municipal solid waste are produced annually worldwide, with large quantities often disposed of in unauthorized areas [1]. This poses significant risks to both the environment and human health. These unregulated dumpsites contribute to a variety of health issues, including respiratory diseases, cardiovascular problems, mental health concerns, and other conditions [2,3]. Rapid urbanization has made waste management a challenge for local authorities in many LMICs. In some regions, up to 50% of collected waste ends up in uncontrolled landfills [4]. Illegal dumping often results from community negligence, lack of awareness about proper waste disposal, inadequate waste storage facilities, inconsistent waste collection schedules, and high municipal service costs. Poorly managed municipal solid waste systems exacerbate the problem, leading to widespread dumping in open fields, roadsides, and riverbanks [2,4,5]. Despite global policies on waste disposal, many remain unenforced, impacting both human and environmental health [6].

Illegal waste dumping releases harmful gases and particles into the environment and promotes the spread of vector-borne diseases, particularly through stagnant water that attracts mosquitoes and other insects [7]. Proximity to waste dumps has been linked to a higher prevalence of respiratory symptoms. Studies have shown that those living near dumping sites report more frequent respiratory issues compared to those living further away. For example, a study in the United States by Khan et al. [8] found significantly higher rates of wheezing, shortness of breath, and other respiratory symptoms among residents living near dumpsites. Similar findings were reported in Ethiopia, where proximity to landfills was associated with coughing, sneezing, sinus issues, chest pains, and colds [2]. In Zimbabwe, Munyai et al. (2020) found higher rates of respiratory problems, such as asthma and nasal irritation, among those living near waste dumps [4]. Other studies, such as those by Matoloni et al. [9], also suggest that living within 5 km of a landfill increases the risk of respiratory diseases and even mortality. Vulnerable populations, including children, pregnant women, and the elderly, are particularly susceptible to these health risks.

In Africa, the WHO estimates that one-third of the disease burden is linked to environmental factors, including poor waste management [10]. In many African cities, less than 20% of waste is disposed of properly, with the remaining 80% ending up in illegal dumpsites [11]. As urbanization and waste generation continue to rise, waste management challenges are expected to worsen. South Africa produces about 12.7 million tons of waste each year. Municipalities are struggling to keep up with the growing amount of waste and provide proper waste management services. Around 3.67 million tons of this waste are not collected or treated properly, leading to large amounts being dumped illegally. This issue is especially noticeable in South Africa’s growing townships and cities. Implementing waste management legislation remains a challenge. Although local governments are responsible for waste management in communities, limited resources, overpopulation, and the presence of informal settlements make effective waste management difficult [12]. For example, a study conducted in the Eastern Cape identified a total of 120 dumpsites. Almost half (45%) were located in green spaces, mainly within urban parks. The rest were found in open areas such as street edges, around public infrastructure, in vacant residential plots, and along the boundaries of public service facilities such as schools and clinics [13].

In South Africa, the National Waste Management Strategy (NWMS) [14] is a legislative mandate under the National Environmental Management: Waste Act, 2008 [15], designed to achieve the Act’s objectives. The strategy addresses the key challenges in South Africa’s waste management sector, such as rising waste volumes driven by population growth, urbanization, and industrialization, as well as the growing complexity of waste streams. It also highlights a historical backlog in waste services, particularly in informal urban and rural areas, where inadequate services contribute to poor living conditions. The lack of reliable waste data further complicates effective management, while the current policy and regulatory framework limits the potential for advancing the waste management hierarchy, stalling sector progress. Furthermore, the absence of recycling infrastructure, outdated waste management facilities, underpricing of waste services, and insufficient compliant landfills and hazardous waste facilities present major obstacles. The strategy is structured around eight primary goals: promoting waste minimization and recycling, ensuring efficient waste service delivery, increasing the waste sector’s contribution to the green economy, raising awareness of the health and environmental impacts of waste, achieving integrated waste management planning, ensuring effective financial management of waste services, remediating contaminated land, and ensuring compliance with the Waste Act. However, the practical implementation of this strategy remains a significant challenge [16].

Strengthening the regulatory framework is, therefore, crucial for improving waste management outcomes. Lessons from other contexts, such as Portugal, demonstrate the potential benefits of regulatory reform. Marques and Simões (2008) [17] describe how the establishment of a governance model and a national regulatory authority in Portugal led to measurable improvements in the quality of urban waste services. A key feature was the adoption of “sunshine regulation”, a benchmarking system that publicly disclosed performance indicators of urban waste utilities. Despite limited coercive power, this “name and shame” approach promoted transparency and incentivized service providers to improve performance. While South Africa’s NWMS outlines ambitious goals, its success depends on translating these into actionable policies supported by clear regulation, consistent monitoring, and institutional accountability. Adopting adaptive and transparent regulatory mechanisms could help overcome implementation gaps and drive progress toward sustainable waste management.

Sustainable Development Goal 11 (SDG 11) calls for inclusive, safe, and resilient cities. However, ongoing challenges in waste management practices and service access continue to hinder progress towards achieving this goal. This study aimed to investigate the risk factors associated with acute respiratory symptoms among communities living near open illegal domestic waste dumping sites and to explore community perceptions regarding illegal dumping of waste. To achieve this, the paper is organized as follows: the next section provides a detailed description of the study design, area and population, selection of participants, data collection, and analysis. The Results section presents the findings on the prevalence of acute respiratory symptoms, associated risk factors, and the community’s perceptions of illegal dumping. The Discussion interprets these findings in relation to the existing literature and policy implications. Finally, the paper concludes with key recommendations for waste management interventions and public health protection in affected communities.

## 2. Materials and Methods

### 2.1. Research Design, Study Setting, and Population

**Figure 1:**
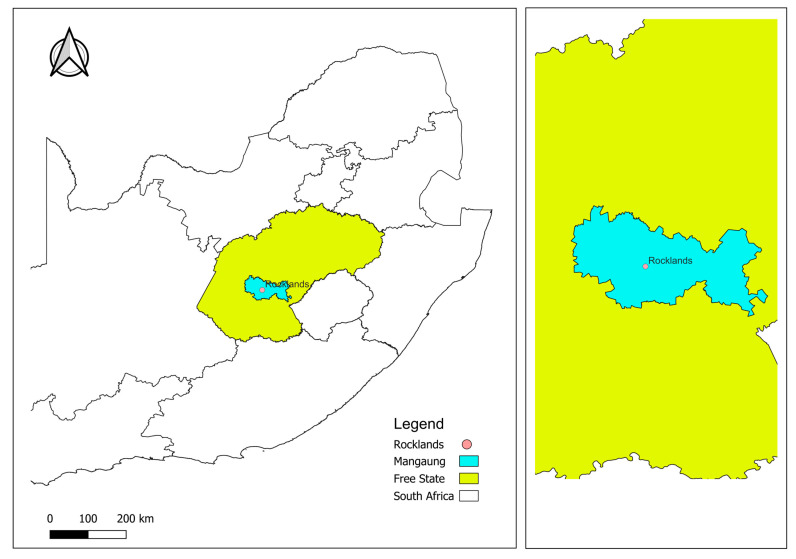
Map of the study area: Rocklands, Mangaung Metropolitan Municipality.

This study employed a quantitative, descriptive cross-sectional design. It was conducted in Rocklands, Bloemfontein, an area characterized by three illegal waste dumping sites located on open land along a single street. The closest households to the dumping sites are within 100 m. The housing in the area consists of bond houses and four-room houses provided by the Mangaung Housing Department. The population is primarily composed of Black African (56.1%), Colored (12.8%), Indian (0.8%), and White (29.8%) residents. The most commonly spoken languages are Sotho (47%), Setswana (27%), and Xhosa (16%). According to the 2011 Census, Rocklands has a population of 48,676 people living in 16,238 households [18]. The map of the study area is shown in Figure 1 below.

### 2.2. Selection of Houses and Study Sample

A systematic sampling method was used to select households, beginning with a random starting point and a fixed periodic interval. Every second household was chosen until the target sample of 200 participants was reached, consisting of 117 individuals with high exposure (living ≤ 5 km from waste dumping sites) and 83 individuals with lower exposure (living > 5–10 km from waste dumping sites). The head of the household was initially approached to participate, and if unavailable, an adult household member willing to participate was invited. The inclusion criteria required participants to be 18 years or older, have lived in the study area for more than one year, and reside within 10 km of a dumping site.

### 2.3. Data Collection

We used a questionnaire adapted from the European Community Respiratory Health Survey (ECRHS-II), which included questions about wheezing or whistling in the chest, feelings of chest tightness, and instances of being woken up by a coughing attack, all within the previous 12 months (March 2021 to March 2022). Additionally, sociodemographic data such as age, sex, race, education, income, and duration of stay were collected. To understand the community’s perceptions, knowledge, and attitudes towards illegal dumping of waste, the questionnaire included a series of closed and open-ended questions designed to assess the participants’ awareness of the problem, their understanding of the associated health risks, and potential strategies to address illegal dumping. The original English questionnaires were translated into Setswana, Sesotho, and Xhosa prior to data collection.

### 2.4. Statistical Analysis

All data were cleaned and error-checked using Microsoft Excel before being exported to Stata IC version 18 (StataCorp., College Station, TX, USA) for further analysis. Descriptive statistics, including frequency distributions and percentages, were used to summarize sociodemographic characteristics, community perceptions, and respiratory symptoms. Chi-squared tests were conducted to assess the associations between dependent and independent variables. We used generalized linear models (GLM) with a log link and Poisson distribution to estimate the prevalence ratios (PRs) and their 95% confidence intervals (CIs) for the association between the proximity to illegal dumping sites and the prevalence of self-reported respiratory symptoms. Generalized linear models (GLMs) are well-suited for studies assessing the association between respiratory symptoms and proximity to landfill sites due to their flexibility in handling various types of outcome variables commonly encountered in health research. For instance, when an outcome is binary (e.g., presence or absence of symptoms such as coughing or wheezing), GLMs with a logit or probit link function allow for accurate modeling of the probability of symptom occurrence [19]. This approach is appropriate for modeling binary outcomes in cross-sectional data where the outcome is common, as it provides direct estimates of PRs rather than odds ratios, which can overestimate risk when the outcome is not rare.

The general form of GLMs used was as follows:

Log (E[*Yi*]) = β_0_ + β_1_ × Proximity*_i_* + β_2_ × Covariates*_i_*

where *Yi* is the binary outcome variable for individual *i* (e.g., presence of wheezing = 1, absence = 0), E[*Yi*] is the expected value of the outcome (i.e., the prevalence), Proximity*_i_* is an indicator variable representing the distance to an open illegal dumping site (<5 km vs. >5 km [reference]), and Covariates*_i_* include potential confounders such as sex, age, education level, and duration of exposure depending on the model.

We built our main model using a stepwise approach, progressively adjusting for confounders in successive models. Model one (M1) was the baseline model, adjusting for sex, age, and population group. Model two (M2) was M1 further adjusted for educational attainment. Model three (M3) was our main model, which incorporated all the adjustments made in M2 and duration of stay. We performed stratified analyses based on sociodemographic characteristics, including age, gender, and education level, to examine whether the relationship between respiratory symptoms and proximity to illegal dumping sites varied across these groups. We present our effect estimates as prevalence ratios (PRs) with their 95% confidence intervals (95% CIs).

## 3. Results

### 3.1. Sociodemographic Characteristics

Table 1 below displays the descriptive characteristics of the study participants. The majority (65.0%) of the participants were aged between 18 and 35 years, with more than half (52.0%) being male. The most frequently spoken languages were Sesotho (39.0%) and Setswana (34.0%). In terms of income, the largest group (42.0%) earned between R500 and R5000. Regarding educational level, almost 80% of the participants had completed Grade 12 or higher (higher certificate or undergraduate).

### 3.2. Prevalence of Respiratory Symptoms

The prevalence of respiratory symptoms reported over the previous twelve months was as follows: wheezing or whistling in the chest (15.0%), chest tightness (15.0%), shortness of breath (21.0%), and cough (37.0%). Results of the χ^2^ test for the association between respiratory symptoms and demographic characteristics are shown in Table 2. Respiratory symptoms were more prevalent among those living within 0–5 km of an illegal dumping site, particularly for wheezing or whistling in the chest (20% vs. 7%, *p* < 0.05), chest tightness (20% vs. 7%, *p* < 0.05), and coughing (43% vs. 27%, *p* < 0.05). The >51-year-old age group had a higher prevalence of wheezing or whistling in the chest (31.0%), shortness of breath (58.0%), and coughing (58%) compared to younger age groups (*p* < 0.05). A longer duration of stay (5+ years) was associated with higher rates of wheezing or whistling in the chest (16.0% vs. 7.0%), chest tightness (15.0% vs. 11.0%), shortness of breath (23.0% vs. 8.0%), and coughing (37.0% vs. 32.0%) compared to 1–4 years of exposure, though these differences were not significant (Table 2).

### 3.3. Generalized Linear Model Analysis of Proximity to Illegal Dumping Sites and Respiratory Symptoms

Proximity to illegal dumping sites (<5 km) was associated with increased respiratory symptoms. For wheezing, the prevalence ratio (PR) was 2.72 (95% CI: 1.56–6.39), which decreased to 2.56 (95% CI: 1.05–6.24) after adjusting for demographic factors and increased to 2.77 (95% CI: 1.10–6.98) following additional adjustment for exposure duration, with statistical significance maintained across all models. Similarly, chest tightness showed a significant association, with an unadjusted PR of 2.78 (95% CI: 1.18–6.54), which attenuated to 2.73 (95% CI: 1.15–6.52) after demographic adjustments and increased slightly to 2.86 (95% CI: 1.19–6.84) upon adjusting for exposure duration. In contrast, shortness of breath demonstrated no significant association, with PRs ranging from 1.25 (95% CI: 0.70–2.27) in the unadjusted model to 0.81 (95% CI: 0.46–1.43) after full adjustments. For coughing, the unadjusted PR of 1.59 (95% CI: 1.05–2.41) was statistically significant, but became nonsignificant after adjustments for demographic factors (PR: 1.46; 95% CI: 0.93–2.26) and exposure duration (PR: 1.45; 95% CI: 0.92–2.27) (Table 3).

Table 4 presents the relationship between respiratory outcomes and the proximity to landfill sites, stratified by gender, age, and education level. For females, the prevalence of wheezing near a landfill was slightly higher, though not statistically significant. Individuals aged 36–50 years had a significantly higher prevalence of wheezing (PR: 2.05, 95% CI: 0.53–3.56). Those with primary education or up to grade 9 had a higher prevalence of wheezing compared to the individuals with grade 12 or higher education. For chest tightness, a higher prevalence of chest tightness was observed (PR: 1.58, 95% CI: 0.12–3.03). The individuals with primary education or up to grade 9 showed a strong and statistically significant association with chest tightness (PR: 16, 95% CI: 15.43–17.86). No significant association was found for the age groups of 36–50 years (PR: 0.99, 95% CI: −0.70–2.07) or >51 years (PR: 0.92, 95% CI: −0.47–2.32). With regard to shortness of breath and coughing, no significant associations were observed between the groups.

### 3.4. Awareness, Perceptions, and Solutions to Illegal Domestic Waste Dumping

Awareness of illegal waste dumping was high among the participants, with 88.5% recognizing it as a problem in their community, while a smaller proportion (8.5%) disagreed and 2.0% were unsure. However, awareness of the associated negative health effects was notably lower, with only 38.0% of the participants acknowledging the health risks, while 48.0% were unaware and 13.0% uncertain. Regarding the frequency of waste dumping, the majority (58.5%) reported observing waste being dumped daily, 23.5% noticed it weekly, 14.5% hardly ever observed dumping, and 3.5% were unsure.

The participants identified adults aged 20–54 as the primary group responsible for illegal waste dumping (62.0%), with teenagers (18.0%) and a combination of teenagers and adults (12.0%) also contributing. Most participants (80.0%) believed the issue had persisted for many years, though 14.0% disagreed and 7.0% were uncertain. The primary causes of illegal dumping were identified as inadequate municipal services (40.5%), open spaces (28.0%), the combination of both factors (22.0%), and lack of knowledge about proper waste disposal (7.0%). Additionally, 22.0% of the participants admitted to engaging in illegal dumping, while 78.0% reported no involvement.

Community-driven initiatives were widely supported by the participants as effective measures to address illegal waste dumping. Awareness campaigns received strong backing, with 91.0% believing that educating the public on proper waste disposal and the harms of illegal dumping would be impactful, while only 4.0% disagreed and 5.0% were unsure. Recycling was favored by 32.0% of the participants, 26.0% emphasized the importance of maintaining community cleanliness, and 23.0% supported a combination of actions, such as stopping dumping and reporting violators. Additionally, 94.0% of the participants expressed a willingness to sort waste if provided with separate bins, while 2.0% were unwilling, and 4.0% remained unsure (Table 5).

## 4. Discussion

This study investigated the risk factors associated with acute respiratory symptoms among the communities residing near open illegal domestic waste dumping sites in Rocklands, Bloemfontein, South Africa, and explored community perceptions of illegal waste dumping. We found a significant association between the proximity to illegal dumping sites and respiratory symptoms, particularly wheezing and chest tightness, as reported elsewhere [4,19,20,21]. Significant associations were found for individuals aged 36–50 years (wheezing) and those with primary education or up to grade 9 (wheezing and chest tightness). Most participants perceived illegal dumping as a significant problem in their community. They reported frequently observing daily waste dumping, attributing this issue primarily to inadequate municipal services and the availability of open spaces. Participants expressed their support for interventions such as recycling programs and educational campaigns, highlighting these as potential solutions to address the issue.

Rapid urbanization and economic expansion have resulted in an increase in environmental hazards due to improper waste management. According to the World Health Organization, improper waste management is a major health threat and has been reported to be associated with a variety of diseases, including cardiovascular diseases, adverse birth outcomes, and respiratory diseases. Vulnerable populations, such as pregnant women, the elderly, and children, are at an increased risk of developing adverse health problems [1]. In this study, respiratory symptoms were more prevalent among those living within 0–5 km of an illegal dumping site, particularly wheezing (20% vs. 7%, *p* < 0.05), chest tightness (20% vs. 7%, *p* < 0.05), shortness of breath (23% vs. 18%, *p* < 0.05), and coughing (43% vs. 27%, *p* < 0.05). In our multivariate models, the proximity to illegal dumping sites (<5 km) was significantly associated with wheezing (PR: 2.77; 95% CI: 1.10–6.98) and chest tightness (PR: 2.86; 95% CI: 1.19–6.84). This aligns with findings from studies conducted in Italy, Malaysia, the USA, Zimbabwe, South Africa, and other regions, which demonstrated increased risks of respiratory morbidity among populations living near waste sites, including elevated risks of asthma and other respiratory illnesses within similar proximities [4,19,20,21]. For example, in a study conducted in Malaysia, 975 schoolchildren aged 6–17 years attending five public schools within a 5 km radius were reported to have symptoms such as breathing difficulties, cough, eye and throat irritation, nausea, and vomiting, with inhalation being the most likely route of exposure [22]. In another study conducted by Tomita et al. [19], in South Africa, living within 5 km of a waste site was significantly linked to an increased risk of asthma (adjusted relative risk: 1.41; 95% CI: 1.20–1.64), tuberculosis (1.18; 1.02–1.36), diabetes (1.25; 1.05–1.49), and depression (1.08; 1.03–1.14). In this study, no significant association was observed between respiratory symptoms, specifically coughing and shortness of breath, and the proximity to illegal dumping sites. The absence of a significant relationship, after adjusting for potential confounders, may be attributable to several factors, including the possibility that other unmeasured confounding variables could influence these outcomes [23].

In this study, sociodemographic characteristics and duration of stay did not appear to influence the respiratory symptoms observed among the participants. However, after stratification, respiratory symptoms were more prevalent among the individuals aged 36–50 years (wheezing) and those with primary education or grade 9 (wheezing and chest tightness). These findings are in line with studies conducted elsewhere. For example, a study conducted in India reported a more than threefold increased likelihood of respiratory illness among individuals above 40 years old (OR: 3.35, CI: 1.61–6.96) compared to younger groups. In the same study, education had a protective effect, with those having more than 5 years of education being less likely to experience respiratory symptoms compared to the illiterate group (OR: 0.44, CI: 0.22–0.91). Additionally, individuals who had stayed in the area for more than 20 years were nearly twice as likely to experience respiratory illnesses compared to the reference group (OR: 1.92, CI: 1.02–3.59) [24]. However, we did not observe any association with other respiratory symptoms, such as coughing and shortness of breath, after stratifying by age, gender, and education. The lack of association in our study may be attributed to the relatively small sample size, which can limit the statistical power of the analysis, making it challenging to detect subtle differences or relationships [24]. Furthermore, the low variation between groups may have further reduced the ability to observe significant effects.

Regarding community perceptions, 88.5% of the participants were aware of illegal dumping in their community, but only 38% recognized the associated health risks. The perceptions of the participants with regard to the causes of illegal dumping included inadequate municipal services and open spaces. Community-driven initiatives, including awareness campaigns and recycling, were strongly supported, with 94.0% willing to sort waste if provided with separate bins. Overall, our findings highlight the importance of public health campaigns and suggest the necessity of integrating environmental education into community outreach programs. This aligns with findings from a review on participatory research, which emphasized the role of community engagement in addressing waste management issues [25].

Community attitudes toward waste disposal in LMIC areas reflect a broader trend observed in similar settings globally, where inadequate infrastructure, unemployment, and spatial limitations contribute to illegal dumping. Niyobuhungiro and Schenck [26] found that 32% of participants linked illegal dumping to the absence of shared waste containers, while poverty and unemployment created a sense of apathy and helplessness. Lorren et al. [27] similarly reported that residents in high-density, low-income areas with limited indoor space often resort to dumping waste outside to maintain clean living conditions. These attitudes are shaped not only by individual behavior, but also by systemic issues such as irregular municipal collection and unequal service delivery between formal and informal settlements. High-income countries typically exhibit stronger community engagement and environmental awareness, supported by regular services, public education campaigns, and established recycling systems.

To address this gap, communities can play a more proactive role in preventing illegal dumping and mitigating its health and environmental impacts. The community’s expressed willingness to engage in sorting waste if provided with the necessary infrastructure, such as separate bins, highlights an opportunity for local governments to promote community-based waste management programs. These can be complemented by public health education campaigns to raise awareness about the health risks of poor waste handling and encourage behavior change. By integrating community-driven strategies into waste management, exposure to harmful pollutants can be reduced, leading to better public health outcomes. Policymakers can use the findings of this study to guide targeted interventions at both municipal and national levels by focusing on improving waste management infrastructure, enhancing waste disposal services, enforcing stricter regulations on illegal dumping, and improving public health surveillance [6]. The study highlights the need to evaluate the effectiveness of such interventions, which can inform evidence-based policies and resource allocation. At the national level, these insights can contribute to broader environmental health strategies and inform regulations on waste management practices.

Strengthening the regulatory environment is also essential for improving waste management outcomes. Drawing from international examples, such as the regulatory reforms in Portugal, offers valuable insights into potential benefits. Marques and Simões [17] highlight how the establishment of a governance model and a national regulatory authority in Portugal led to notable improvements in urban waste services. To address the challenges of implementation and ensure progress towards sustainable waste management, adopting adaptive and transparent regulatory mechanisms is crucial.

Additionally, local policy enforcement plays a critical role in preventing illegal dumping by ensuring compliance with waste management regulations and holding violators accountable. Weak enforcement due to limited resources, lack of political will, or inadequate monitoring can contribute to the persistence of illegal dumping, particularly in low-income areas. Strengthening enforcement requires a multifaceted approach, including clearer regulations, increased funding for inspection and monitoring, public reporting mechanisms, and stronger penalties for non-compliance. In addition, community involvement in monitoring and reporting illegal dumping, combined with public education and access to proper waste disposal services, can enhance enforcement efforts and foster a sense of shared responsibility [28].

In light of emerging technologies identified in recent circular economy research, such as digital waste tracking systems, smart bins, and life cycle assessment tools, municipalities can enhance the design, implementation, and monitoring of waste management programs. For example, integrating technologies such as the Internet of Things (IoT) and machine learning can help optimize waste collection routes, detect illegal dumping in real time, and monitor community compliance. These innovations support data-driven decision-making and help allocate resources more efficiently [29]. Our findings align with several objectives of the National Waste Management Strategy (NWMS), such as promoting waste minimization and improving waste service delivery. However, the challenges identified in this study—such as illegal dumping and lack of infrastructure—highlight significant barriers that the NWMS also addresses. The gaps in public awareness and effective waste management suggest that the current approach may need further adaptation to better address the health risks identified in our study and meet the needs of affected communities.

The study’s strength lies in its focus on an underresearched population in a low-income setting. Thus, this research contributes meaningfully to the growing body of evidence on environmental health disparities in LMICs and aligns with global public health priorities aimed at improving waste management and mitigating the adverse health impacts of environmental exposure in vulnerable communities. The application of GLMs, adjusting for potential confounding variables, enhanced the analytical rigor and allowed for more robust estimation of associations between exposure and health outcomes. The integration of both self-reported respiratory symptoms and community perceptions regarding the prevalence, causes, and consequences of illegal dumping highlights the environmental justice implications for affected populations and also enhances the policy relevance of the findings.

However, this study had several limitations. The use of a cross-sectional design prevented the establishment of causality, as exposure and outcomes were measured at the same time. Without knowing whether exposure preceded the onset of symptoms, we cannot infer a causal link. A longitudinal study design, which follows participants over time, would allow for the assessment of changes in exposure and the development of respiratory outcomes.

The survey only relied on self-reported responses regarding respiratory problems, without any objective medical assessment to confirm the presence of actual respiratory conditions. As a result, the findings are based on the respondents’ perceptions of respiratory issues rather than clinically verified diagnoses. To draw more definitive conclusions, it would be essential to cross-check the survey responses with medical records or perform clinical evaluations such as spirometry tests to confirm the existence of respiratory problems.

Another key limitation of this study is the classification of respondents based solely on their proximity to an illegal landfill (0–5 km vs. >5 km). While this approach provides an initial assessment of potential exposure, it does not account for other environmental factors that may contribute to respiratory health outcomes. Additionally, the 5 km threshold for exposure was determined based on the existing literature. However, it is possible that alternative distance thresholds could have provided additional insights, as factors such as meteorological conditions may influence the relationship between the proximity to dumping sites and health outcomes.

A larger sample size would enhance the reliability and accuracy of the study findings by increasing statistical power and improving the detection of true associations. It would also allow for more effective control of confounding variables and enable meaningful subgroup analyses. These benefits would contribute to greater generalizability and validity of the results. In our study, the limited sample size may have reduced the ability to detect associations between the proximity to landfill sites and certain respiratory symptoms, such as coughing and shortness of breath.

Factors such as heavy traffic, industrial facilities, energy plants, agricultural practices such as straw burning, tobacco smoking, and the use of wood for cooking and heating may also influence respiratory symptoms in the study population. The absence of data on these factors in the current study is a key limitation, as we were unable to account for them in our models, thereby limiting our ability to attribute the observed health effects solely to landfill proximity. Future research should incorporate comprehensive assessments to strengthen causal inferences.

Environmental assessments were not conducted in this study. Assessing environmental pollutants and seasonal variations in concentrations would have provided insights into the types of pollutants present and their association with respiratory outcomes. Future research should focus on measuring pollutants at the personal, household, and community levels across seasons to provide a more precise estimate of the effect of environmental exposure on respiratory symptoms. Future studies should incorporate comprehensive environmental assessments, including air quality monitoring and geospatial analysis, to better distinguish the relative contributions of multiple pollution sources. The effectiveness of various interventions, such as public health education campaigns, improving waste management, or community-level waste sorting programs and potential barriers to waste sorting and recycling, need to be explored.

## 5. Conclusions

This study highlights the significant health risks associated with the proximity to illegal domestic waste dumping sites, particularly in communities near Rocklands, Bloemfontein. The prevalence of acute respiratory symptoms such as wheezing, cough, chest tightness, and shortness of breath was notably higher among the individuals living within 5 km of the waste sites, with statistically significant associations observed for wheezing and chest tightness. These findings highlight the urgent need for improved waste management and public health interventions. Despite the recognition of illegal dumping as a persistent environmental problem, awareness of its health consequences remains low, with only 38% acknowledging the associated risks. Community education efforts, alongside enhanced waste collection services, recycling initiatives, and public awareness campaigns, are critical for mitigating these health impacts. Addressing this problem requires a comprehensive approach that includes not only infrastructural improvements, but also efforts to raise awareness and change community behaviors surrounding waste disposal. Strengthening the regulatory framework is vital for improving waste management outcomes. To ensure progress toward sustainable waste management, South Africa must adopt adaptive, transparent regulatory mechanisms that address implementation challenges effectively. Thus, addressing the challenges related to waste management requires substantial investments in waste management infrastructure, including the provision of shared waste containers, reliable waste collection services, and space for waste storage in high-density areas. Moreover, investments in public education and community engagement are essential to shift attitudes and encourage more responsible waste disposal practices.

## Figures and Tables

**Table 1:** Descriptive statistics of the sociodemographic characteristics of the participants.

Characteristics		*N* (%)
Age	18–35	126 (65.0)
	36–49	51 (22.0)
	50–65	23 (13.0)
Ethnicity	Black	200 (100)
Gender	Male	104 (52.0)
	Female	92 (46.0)
	Binary	4 (2.0)
Home language	Setswana	67 (34.0)
	Sesotho	78 (39.0)
	Xhosa	50 (25.0)
	Zulu	5 (2.0)
Income group Conversion date: 7 April 2025 (Rands to USD)	$27–$270	84 (45.0)
	$270–$540	42 (22.0)
	$540–$1080	15 (8.0)
	Do not want to answer	46 (25.0)
Educational level	Primary/Grade 9	41 (21.0)
	Grade 12/higher certificate/ undergraduate	159 (79.0)
How long have you stayed in this area?	1–4 years	27 (13)
	5 years and more	173 (87.0)

**Table 2:** Prevalence of acute respiratory symptoms by sociodemographic characteristics, duration of stay, and distance to an illegal dumping site.

	Distance		Age			Gender		Education		Income		Duration	
Variable	0–5 km	>5 km	18–35 Years	36–50	>51	Female	Male	Primary/Gr 9	Gr 12/Higher Certificate/ Undergraduate	≤R10,000	>R10,000	1–4 Years	5 and More
Wheezing or whistling in the chest	23 (20.0)	6 (7.0) *	13 (10)	8 (18)	8 (31) *	15 (15)	14 (15)	5 (12)	24 (15)	13 (10)	14 (23) *	2 (7)	27 (16)
Chest tightness	23 (20.0)	6 (7.0) *	15 (12)	9 (21)	5 (19)	18 (18)	11 (12)	5 (13)	24 (16)	13 (10)	13 (22) *	3 (11)	26 (15)
Shortness of breath	24 (23)	14 (18)	11 (9)	12 (31)	15 (58) *	20 (22)	17 (20)	14 (35)	24 (17) *	26 (22)	11 (21)	2 (8)	36 (23)
Coughing	48 (43)	22 (27) *	35 (28)	20 (48)	15 (58) *	36 (36)	33 (38)	15 (39)	55 (36)	38 (31)	30 (52) *	8 (32)	62 (37)

* *p* < 0.05.

**Table 3:** Prevalence ratios (PRs) and their 95% confidence intervals (CIs) with increasing adjustment for the association between the proximity to open illegal dumping sites and respiratory symptoms.

Variable	M1 *	M2 *	M3 *
	PR (95% CI)	PR (95% CI)	PR (95% CI)
Wheezing or whistling in the chest			
Proximity to open illegal dumping sites			
>5 km (ref.)			
<5 km	2.78 (1.56–6.39) **	2.56 (1.05–6.24) **	2.77 (1.10–6.98) **
Chest tightness			
Proximity to open illegal dumping sites			
>5 km (ref.)			
<5 km	2.78 (1.18–6.54) **	2.73 (1.15–6.52) **	2.86 (1.19–6.84) **
**Shortness of breath**			
Proximity to open illegal dumping sites			
>5 km (ref.)			
<5 km	1.25 (0.70–2.27)	0.80 (0.46–1.38)	0.81 (0.46–1.43)
**Coughing**			
Proximity to open illegal dumping sites			
>5 km (ref.)			
<5 km	1.59 (1.05–2.41)	1.46 (0.93–2.26)	1.45 (0.92–2.27)

Note: * M1: unadjusted model; M2: adjusted for sex, age, and education; M3: adjusted for M2 and the duration of exposure; ** prevalence ratios (PR) were considered statistically significant at the 95% confidence interval (CI).

**Table 4:** Prevalence ratios (PRs) and 95% confidence intervals (CIs) for the association between the proximity to open illegal dumping sites and respiratory symptoms, stratified by age, gender, and education level.

Outcome	Sociodemographic Characteristics	PR (95% CI)
Wheezing	Female	1.37 (−0.08–2.81)
	Male	0.73 (−0.73–2.19)
	36–50	2.05 (0.53–3.56) *
	>51	0.07 (−0.88–1.02)
	Primary/Gr 9	17.89 (16.97–18.81) *
	Gr 12/higher certificate/undergraduate	0.84 (−0.14–1.80)
Chest tightness	Female	0.80 (−0.26–1.86)
	Male	1.58 (0.12–3.03)
	36–50	0.99 (−0.70–2.07)
	>51	0.92 (−0.47–2.32)
	Primary/Gr 9	16 (15.43–17.86) *
	Gr 12/higher certificate/undergraduate	0.85 (−0.05–1.75)
Shortness of breath	Female	−0.27 (−0.96–0.42)
	Male	−0.03 (−1.25–1.20)
	36–50	0.35 (−0.99–1.70)
	>51	−0.49 (−1.08–0.09)
	Primary/Gr 9	−0.38 (−1.15–0.39)
	Gr 12/higher certificate/undergraduate	0.56 (−0.88–0.92)
Coughing	Female	0.28 (−0.32–0.88)
	Male	0.48 (−0.27–1.28)
	36–50	0.54 (−0.08–1.16)
	>51	0.15 (−0.44–0.74)
	Primary/Gr 9	−0.49 (−1.50–0.51)
	Gr 12/higher certificate/undergraduate	0.47 (−0.02–0.98)

Note: * prevalence ratios (PR) were considered statistically significant at the 95% confidence interval (CI).

**Table 5:** Awareness, perceptions, and proposed solutions to illegal domestic waste dumping (n = 200).

Characteristics		*N* (%)
Do you think illegal dumping of domestic waste is a problem in your community?	No	17 (8.5)
	Yes	177 (88.5)
	Do not know	4 (2.0)
	Missing	2 (1.0)
Are you aware of the negative health effects of illegal domestic waste dumping?	No	93 (48.0)
	Yes	76 (38.0)
	Do not know	26 (13.0)
	Missing	5 (1.0)
How often do you notice people dumping waste in your area?	I hardly see items being dumped	29 (14.5)
	Everyday	117 (58.5)
	Once a week	47 (23.5)
	I do not know	7 (3.5)
Which age group usually dumps domestic waste in this area?	Children of 2–9 years only	4 (2.0)
	Teenagers of 10–19 years only	35 (18.0)
	Children (2–9 years) and teenagers (10–19 years)	1 (1.0)
	Adults of 20–54 years only	120 (62.0)
	Elderly of 55 years and above	13 (7.0)
	Teenagers and adults	24 (12.0)
	Children and adults	1 (1.0)
	Missing	2 (1.0)
Have you or any family member ever left any item from your household in open spaces?	No	138 (69.0)
	Yes	40 (20.0)
	Missing	22 (11)
Has illegal domestic dumping been a problem in this community in previous years?	No	27 (13.5)
	Yes	159 (79.5)
	Do not know	13 (6.5)
	Missing	1(0.5)
What are the causes of illegal domestic waste dumping in your community?	Multiple open spaces	57 (28.5)
	Affordable	1 (0.5)
	Lack of knowledge regarding proper disposal of waste	14 (7.0)
	Lack of municipal services	80(40.0)
	Multiple open spaces and lack of municipal services	43 (21.5)
	Multiple open spaces and lack of knowledge regarding disposal of waste	4 (2.0)
	Missing	1 (0.5)
What are the measures which can be implemented by the community to reduce the issues of illegal domestic waste dumping?	Stop dumping waste	20 (10.0)
	Report a person dumping	18 (9.0)
	Keep the community clean	51 (25.5)
	Recycling	64 (32.0)
	All of the above	45 (22.5)
	Missing	2 (1.0)
Can awareness campaigns which are related to proper waste disposal and education on the effects of illegal waste dumping be of value?	No	8 (4.0)
	Yes	181 (90.5)
	Do not know	10 (5.0)
	Missing	1 (0.5)
If there are different bins for each type of waste allocated to your household, would you be willing to sort it out and dispose of waste correctly?	No	3 (1.5)
	Yes	188 (94.0%)
	Do not know	8 (4.0%)
	Missing	1 (0.5)

## Data Availability

The data from this study are available upon request from the corresponding author, subject to ethical restrictions.
